# Differential Expression of microRNAs in Thymic Epithelial Cells from *Trypanosoma cruzi* Acutely Infected Mice: Putative Role in Thymic Atrophy

**DOI:** 10.3389/fimmu.2015.00428

**Published:** 2015-08-21

**Authors:** Leandra Linhares-Lacerda, Cintia Cristina Palu, Marcelo Ribeiro-Alves, Bruno Diaz Paredes, Alexandre Morrot, Maria Rosa Garcia-Silva, Alfonso Cayota, Wilson Savino

**Affiliations:** ^1^Laboratory on Thymus Research, Institute Oswaldo Cruz, Oswaldo Cruz Foundation, Rio de Janeiro, Brazil; ^2^HIV/AIDS Clinical Research Center, National Institute of Infectious Diseases, Oswaldo Cruz Foundation, Rio de Janeiro, Brazil; ^3^The National Institute of Science and Technology for Structural Biology and Bioimaging, Federal University of Rio de Janeiro, Rio de Janeiro, Brazil; ^4^Department of Immunology, Microbiology Institute, Federal University of Rio de Janeiro, Rio de Janeiro, Brazil; ^5^Functional Genomics Unit, Institut Pasteur de Montevideo, Montevideo, Uruguay

**Keywords:** Chagas disease, thymus atrophy, thymic epithelial cell, microRNA, thymocyte migration

## Abstract

A common feature seen in acute infections is a severe atrophy of the thymus. This occurs in the murine model of acute Chagas disease. Moreover, in thymuses from *Trypanosoma cruzi* acutely infected mice, thymocytes exhibit an increase in the density of fibronectin and laminin integrin-type receptors, with an increase in migratory response *ex vivo*. Thymic epithelial cells (TEC) play a major role in the intrathymic T cell differentiation. To date, the consequences of molecular changes promoted by parasite infection upon thymus have not been elucidated. Considering the importance of microRNA for gene expression regulation, 85 microRNAs (mRNAs) were analyzed in TEC from *T. cruzi* acutely infected mice. The infection significantly modulated 29 miRNAs and modulation of 9 was also dependent whether TEC sorted out from the thymus exhibited cortical or medullary phenotype. *In silico* analysis revealed that these miRNAs may control target mRNAs known to be responsible for chemotaxis, cell adhesion, and cell death. Considering that we sorted TEC in the initial phase of thymocyte loss, it is conceivable that changes in TEC miRNA expression profile are functionally related to thymic atrophy, providing new clues to better understanding the mechanisms of the thymic involution seen in experimental Chagas disease.

## Introduction

The thymus is a common target organ in infectious diseases (1). This primary lymphoid organ is responsible for bone marrow-derived T cell precursors differentiation from the most immature CD4^−^CD8^−^ phenotype to CD4^+^CD8^+^ and finally in CD4^+^CD8^−^ or CD4^−^CD8^+^ T cells that will colonize secondary lymphoid organs (2). These maturation steps occur while these cells migrate through the thymic lobules and interact with microenvironmental cells, particularly thymic epithelial cells (TEC) (3). TEC guide the T cell maturation by production of cytokines, chemokines, hormones, adhesion molecules, extracellular matrix (ECM) proteins, and by expression of different ligands, like Notch, as well as self-peptides in the context of major histocompatibility complex (MHC). The self-peptide presentation determines T cell fate through positive and negative selection events, where immature lymphocytes expressing randomly rearranged T-cell receptor will be selected based on their differential ability to recognize the complex peptide/MHC (4–7). All intrathymic T cell maturation steps generate lineage committed and self-tolerant T cells capable to perform immunological functions in the periphery. However, the intrathymic homeostasis is disrupted in numerous acute infectious diseases leading to thymus atrophy (1). The transient thymic involution can be caused not only by infection but also due to other forms of stress and also occurs progressively with aging in a permanent way, as reviewed elsewhere (8). The biological advantages of thymic involution are currently uncertain, although there is evidence that thymic alterations triggered by *Trypanosoma cruzi* infection explain part of the clinical outcomes observed in chagasic patients (8, 9).

Chagas disease acute phase is characterized by apparent circulating parasites and tissue parasitism with intense production of reactive nitrogen intermediates, such as nitric oxide (NO) and cytokine release: interleukin (IL)-12, interferon (IFN) γ, tumor necrosis factor (TNF) α by macrophages, natural killer (NK) and T cells, with an activation pattern characterized by a polarized type-I response (10). However, there is also the production of anti-inflammatory cytokines, such as IL-4, IL-10, and transforming growth factor (TGF) β, that together with glucocorticoids (GC) control the immune response (11). Such response plays a role in containing parasite replication in acute phase and influences disease severity during the chronic phase of the infection (12).

*Trypanosoma cruzi* acute infection in mice causes a severe thymic atrophy, which becomes noticeable during early infection and increases progressively in parallel with parasitemia and pro-inflammatory cytokine levels (10). Additionally, even though *T. cruzi* infected cells can be found in the thymus (13, 14), current evidence demonstrates that the organ is mostly affected by systemic effects of the infection (15, 16). Actually, the parasite-associated response goes beyond the immune system with the activation of hypothalamus–pituitary–adrenal axis, resulting in hormonal imbalance that affects intrathymic homeostasis (9, 17). The neuroendocrineimmnune imbalance promotes a massive depletion of immature CD4^+^CD8^+^ T cells, which together with the export of these thymocytes to periphery, trigger thymic atrophy (16). Those intrathymic migratory abnormalities somehow benefit the immature thymocytes to bypass negative selection events, which reinforces the role of TEC in thymic atrophy, since in acutely *T. cruzi* infection, TEC enhanced the deposition of ECM, such as laminin and fibronectin, as well as chemokines, favoring developing T-cell migration (18–21). Nevertheless, the mechanism by which TEC mediate thymic involution remains poorly understood. microRNAs (miRNAs) can be envisioned as one group of candidates. miRNAs are small non-coding RNA molecules that suppress gene expression at the post-transcriptional level, and are fine-tuning regulators of diverse biological processes (22, 23).

In these respect, it has been shown that induction of thymic involution through poli(I:C) treatment is under tight control of miRNA-29a, which regulates interferon-α receptor (IFNαR1) in TEC, resulting in a very sensitive mechanism of thymic atrophy (24). In fact, TEC are programed to reduce functionality and suspend thymopoiesis in response to IFN-α (8). Recent studies suggest that miRNAs are important factors in the maintenance of tissue-restricted antigens expression in medullary TEC (7). Taken together, a molecular regulation of infection-associated thymic involution prompted us to analyze the expression of miRNAs in cortical and medullary TEC from *T. cruzi* acutely infected mice.

## Materials and Methods

### Experimental acute *Trypanosoma cruzi* infection

Male C57BL/6 mice were provided by the Oswaldo Cruz Foundation animal facilities (Rio de Janeiro, Brazil). Five weeks old mice were infected by intraperitoneal injection of 1 × 10^3^ *T. cruzi* (Y strain) trypomastigotes. The parasites were maintained by serial passages in male mice from the same strain, harvested after 7 days post-infection (dpi) through cardiac puncture. The collected blood was harvested in vials containing 200 μl of sodium citrate, centrifuged (1,200 rpm) for 10 min, later the plasma was collected after incubating for 30 min in 37°C and centrifuged (3,000 rpm) during 10 min. The pellet containing parasites was resuspended and the trypomastigote concentration was estimated using Neubauer chamber in order to prepare a solution with 5,000 parasites/ml was prepared. Each mouse was infected with 200 μl of this solution. The uninfected (control) mice were kept under the same conditions through the infection progress.

Parasitemia was estimated for all infected animals by direct microscopic observation of 5 ml blood obtained from the tip of the tail. Initially, 10 mice were infected and the parasitemia was done on the following 6–18 days, once the parasitemia pick was determined, the estimation of circulating trypomastigotes was done solely 8 dpi to confirm that the infection was well succeeded.

All experiments and animal handling were conducted according to the rules prescribed by the official ethics committee for animal research of the Oswaldo Cruz Foundation.

### Analysis of thymocyte subpopulations

Sixteen mice were infected as described above and their thymuses were harvested between 9 and 12 dpi, 4 thymuses plus 1 from control mouse per day (total of 16 acutely Chagas infected mice and 4 controls). The organs were individually squeezed in PBS containing fetal calf serum 10% (Gibco). For analysis of thymocyte subsets, cells were resuspended in mouse serum during 15 min and incubated with specific monoclonal antibodies for 30 min at 4°C in the dark (anti-CD4/APC, anti-CD8/FITC, from BD Pharmingen), followed by washing and analysis on flow cytometer FACS Canto II (BD Biosciences) and using the FACS Diva v6.1.3 software. In order to determine specific fluorescence intensity, the background staining values obtained with fluorochrome-matched IgG isotype controls were subtracted. Thymocytes from 12 to 14 dpi also underwent these procedures, four infected and two control mice each time, in order to confirm that the infection led to thymic atrophy.

The variation of CD4^+^CD8^+^ cells due to infection progression was tested by one-way ANOVA, followed by the Tukey’s honestly significant difference (HSD) *post hoc* test.

### Thymic epithelial cell sorting

Five thymuses from 12 days post-infection mice or control mice were used for TEC isolation procedure, which was performed as described (25) with some modifications. Briefly, thymuses were minced and transferred to round-bottom tubes and agitated in 50 ml of RPMI-1640 for 30 min for initial thymocytes release, after which the remaining tissue was digested with two sequential changes of collagenase/DNAse I solution [50 mg/ml collagenase D (Roche), 1 mg/ml DNAse I (Roche) in RPMI medium] at 37°C for 15 min each, followed by one collagenase/dispase/DNAse I [50 mg/ml collagenase/dispase (Roche), 1 mg/ml DNAse I in RPMI medium] at 37°C for 30 min under continuous stirring. Cells were then centrifuged, pooled, and resuspended in cold EDTA/FACS buffer (5 mM EDTA in PBS with 2% FCS and 0.02% NaN_3_), filtered through 100 μm mesh and counted in Neubauer chamber. Then, anti-CD4 and anti-CD8 Dynabeads (Invitrogen) were added at 500 μl/10^8^ cells according to the manufacturer’s protocols. Remaining cells were then pooled and recovered by centrifugation, washed in EDTA/FACS buffer and 5 × 10^6^ cells dispensed into the wells of a 96-well round-bottomed plate for staining. We incubated the biotinylated anti-mouse I-A[b] primary antibody (BD Pharmingen) for 30 min at 4°C, followed by a wash in 100 μl of EDTA/FACS buffer, after we added the secondary APC-Cy7-conjugated streptavidin and the conjugates: FITC-conjugated UEA-1 lectin (Vector), PerCP conjugated anti-CD45 antibody (clone 30-F11), PE-conjugated anti-Ly51 antibody (clone 6C3), APC-conjugated CD326 antibody (EpCAM, clone D8.8), all from BD Pharmingen. Cells isolated and stained as outlined above were resuspended in EDTA/FACS buffer at 1 × 10^6^ cells/ml. Sorting was performed in a FACS Aria II cell sorter (BD Biosciences). Samples were collected in 50% (v/v) fetal calf serum in RPMI, recovered by centrifugation, counted and analyzed for purity.

### RNA extraction

The sorted population was submitted to RNA extraction using miRNEasy (Qiagen), which allows the isolation of small RNA (with miRNAs) and messenger RNA (mRNA) separately. To allow normalization of sample-to-sample variation in miRNA isolation, cDNA synthesis and real-time PCR, synthetic *Caenorhabditis elegans* miRNA cel-miR-39 (Qiagen) was added as 5 μl of 25 pmol solution to each denatured sample (i.e., after combining the sample with Qiazol) and quantified in all samples with an average recovery ranging from 26 to 36 in crossing point (CP) (Figure S1 in Supplementary Material). After this, we proceeded with other extraction steps following the manufacturer’s instructions. The quantity and quality of RNA were assessed on NanoDrop ND-1000 Spectrophotometer (Thermo Scientific) and 2100 Bioanalyzer (Agilent Technologies) using the small RNA LabChip kit and RNA 6000 nano kit (Agilent Technologies).

### *AIRE* gene expression by quantitative polymerase chain reaction

Gene expression for *AIRE* (Autoimmune regulator gene) and reference genes were carried out using 30 ng of total RNA with SuperScript III kit (Invitrogen) for reverse transcription reaction and FAST SYBR Green Master Mix (Applied Biosystems) and the following primers: *AIRE* (F-GGCAGGTGGGGATGGAATGC and R-TTCAGACGGAGCGTCTCCTGG), *HPRT* (F-TCCCAGCGTCGTGATTAGCGATG and R-GGCCACAATGTGATGGCCTCCC) and *RPL13* (F-CCAAGCAGGTACTTCTGGGCCGGAA and R-CAGTGCGCCAGAAAATGCGGC) for quantitative polymerase chain reaction (qPCR) on Step ONE Plus Fast Real Time PCR System (Applied Biosystems).

### miRNA expression profiling

microRNA (30 ng) was submitted to reverse transcription by poly-A-tailing using RT^2^ miRNA First Strand Kit (Qiagen) as described in the manufacturer’s protocol. We then performed miRNA expression profiling using a custom PCR array plate with 85 miRNA (Qiagen) and RT^2^ SYBR Green qPCR Mastermix (Qiagen) on Step ONE Plus Fast Real Time PCR System (Applied Biosystems). For normalization, we used all five references cel-miR-39, snoRNA142, snoRNA251, Rnu6, and snoRNA20 (Figure S1 in Supplementary Material) after gene expression stability analysis (26).

### Quantitative PCR analysis

The fluorescence accumulation data of real-time RT-PCR reactions of each sample were used to fit four parameters sigmoid curves to represent each amplification curve using the library qPCR (27) for the R statistical package version 3.1.2 (28). The cycle of quantification was determined for each amplification, by the maximum of the second derivative of the fitted sigmoid curve. The efficiency of each amplification reaction was calculated as the ratio between the fluorescence of the cycle of quantification and the fluorescence of the cycle immediately preceding that. The estimated efficiency of each miRNA or gene was obtained by the mean of the efficiencies calculated for each amplification reaction of that precise miRNA or gene. microRNA normalization among the different amplified samples was achieved by the calculation of normalization factors given by the geometric mean of the expression value of all expressed miRNAs in a given sample (26). *AIRE* normalization was done by the geometric mean of the expression value of *HPRT* and *RPL13* reference genes. The comparisons of means of normalized miRNA or AIRE expression values between groups were performed by a non-parametric one-way ANOVA with 1,000 unrestricted permutations, followed by *post hoc* pair-wise comparisons with Bonferroni adjustment by a non-parametric *t*-test also with 1,000 permutations (29). Additionally, false-positive ratios (FDR) were estimated to adjust for multiple comparisons (30). Results were represented in graphs displaying the expression levels mean ± SE. Two-tailed levels of significance ≤0.01, 0.05, and 0.1 were considered as “highly significant,” “significant,” and “suggestive,” respectively.

### Bioinformatics-based enrichment analysis of miRNA targets

To predict miRNA targets, we identified putative target genes based on predictions from five online softwares: *miRanda*^1^, *Microcosm Target*^2^, *miRNAMap*^3^, *miRTarBase*^4^, and *Target Scan*^5^. Any gene was considered a putative target if it was predicted in at least three out of the five predicting software. We then performed a gene set enrichment analysis (GSEA) with putative target genes. A gene set was defined as all putative target genes that share the same ontology based on the gene ontology (GO) database (31). The over representation was assessed with a statistical score based on a hypergeometric test with *p*-values ≤0.001. The Rgraphviz package^6^ was used to illustrate the relationship between putative targets, miRNAs and biological processes, and in calculations of the k-core structures of the input networks using the degree as centrality measure. Graphs follow virtual physical models with low energy configuration, and only vectors containing the maximum core membership for each vertex, equal to 11 or greater, were displayed.

## Results

### Thymic atrophy in *Trypanosoma cruzi* acute infection

**Figure 1:**
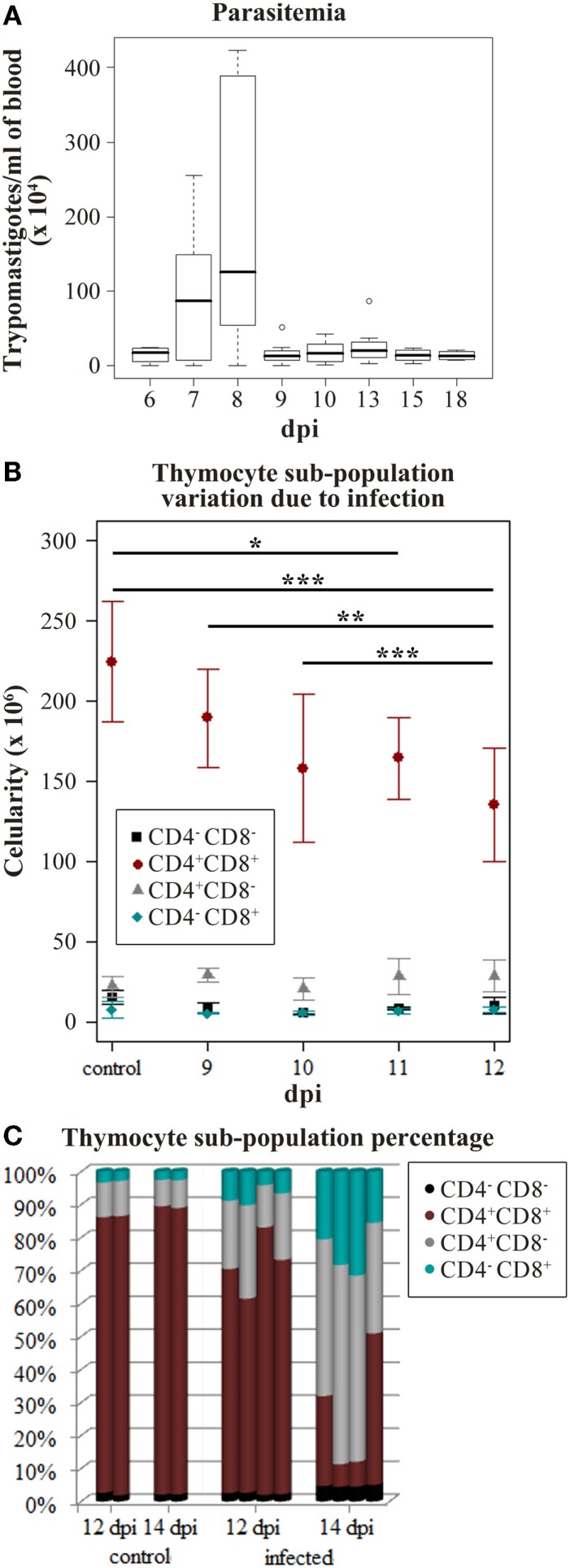
***Trypanosoma cruzi* acute infection induces progressive thymic atrophy and CD4^+^CD8^+^ thymocyte loss**. Nine C57BL/6 mice were infected with 1,000 metacyclic trypomastigotes and in the following 6–18 days the parasitemia was accessed to verify the infection progression. **(A)** The box plot shows the median amount of parasites per blood milliliter, upper and lower quartiles, maximum and minimum values (whiskers) excluding outliers (circles). Once the infection peak was identified (8 dpi), the thymocyte subpopulation variation in the following days was studied in order to identify when the CD4^+^CD8^+^ thymocyte loss starts. **(B)** The cytofluorometric profiles of thymocytes were obtained staining with anti-CD4 and anti-CD8. Thymocytes from three or four mice were submitted to FACS analysis on days 9–12 post-infection (dpi). The plots represent the mean number of cells ±SD in each day. The described decrease on CD4^+^CD8^+^ thymocyte subpopulation due to infection progression was confirmed (ANOVA *p*-value = 0.0002) and the Tukey’s honestly significant difference (HSD) post-test revealed significant differences in each dpi. (adjusted *p*-value ≤*0.05, **0.01, and ***0.001) **(C)** Thymocyte subpopulation percentages data derived from the cytofluorometric profiles show that by 14 dpi the thymic atrophy is reached. Each bar represents thymocytes from a single mouse.

Since the interaction between thymocytes and TEC play a major role in T-cell development, variations in TEC gene expression may alter the thymic environment with consequences on thymocyte fate (4). Accordingly, we analyzed miRNA profiles variation due to infection in the initial point of thymic atrophy to avoid secondary effects caused by thymocyte loss or consequent microenvironmental modifications. We used the decay of CD4^+^CD8^+^ thymocytes number to define when the thymus should be harvested. Intraperitoneal acute infection led to a parasite load picking at 8 dpi, and characterized by the high number of metacyclic trypomastigotes found circulating in the peripheral blood (Figure 1A). During the following days a decrease of CD4^+^CD8^+^ thymocytes was observed (Figure 1B) and later, a severe thymic atrophy with an average loss of 80% of CD4^+^CD8^+^ thymocytes was seen on the 14th dpi (Figure 1C). On the 12th dpi, this cell subpopulation was significantly reduced when compared with cell counting from control and 9–10 dpi mice (Figure 1B), preceding the thymic atrophy, thus we perform the following experiments using samples at this time point of infection.

### Cortical and medullary TEC sorting

**Figure 2:**
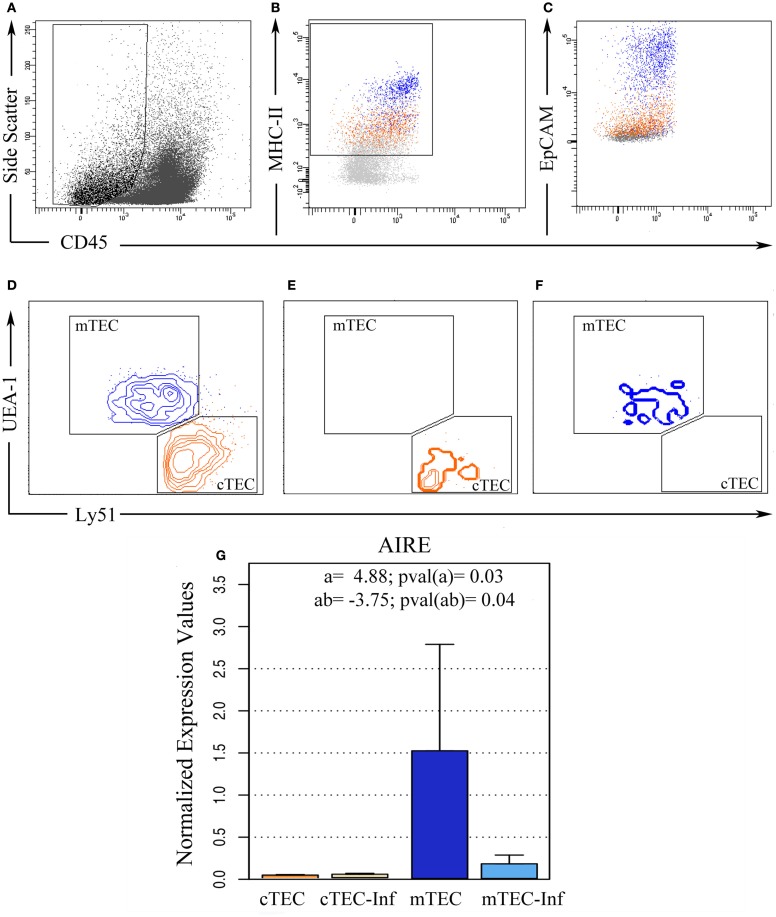
***Ex vivo* thymic epithelial cell sorting**. Five replicates of thymic cell pools from control and infected mice were sorted using flow cytometry in order to isolate TEC. **(A)** Initially, CD45^−^ cells were selected based on size, **(B)** from this population, MHC-II positive cells were isolated after EpCAM confirmation **(C)**. **(D)** Then, according to UEA-1 and Ly51 surface markers, these cells were distinguished between cortical TEC (cTEC, Ly51^+^, and UEA^−^, orange) and medullary TEC (mTEC, Ly51^−^, and UEA^+^, blue) phenotypes. Post-sorting analysis revealed 98% purity in cTEC **(E)** while 95% purity in mTEC **(F)**. **(G)** After miRNA isolation, the remaining mRNA from three experimental pools allowed us to analyze *AIRE* gene expression, confirming if the sorted cells matched the correct expected TEC profile. The bar plot represents the average expression in each condition. “a” indicates the magnitude of the expression ratio (log^−2^) due to TEC phenotype, whereas a positive value shows a higher expression in mTEC. “ab” indicates the expression ratio magnitude (in log^−2^) as consequence of the combination between infection and cell type, whereas a negative value shows that the *AIRE* expression in infected mTEC is lower than in control mTEC.

In order to prepare pure populations of primary (freshly harvested) cortical and medullary TEC (respectively cTEC and mTEC), thymuses from control and infected mice were harvested at 12 dpi and disaggregated by enzymatic digestion, where most thymocytes were eliminated and TEC were enriched, allowing cell sorting. Sorted population was then stained with Ulex europaeus Lectin 1 (UEA1) and antibodies against CD45, MHC-II, EpCAM, and Ly51 and sorted (Figures 2A–D). Post-sort analysis revealed more than 98% purity for cortical TEC (CD45^−^MHCII^+^EpCAM^+^Ly51^+^UEA1^−^) and 95% for medullary TEC (CD45^−^MHCII^+^EpCAM^+^Ly51^−^UEA1^+^) populations (Figures 2E–F, respectively).

To further validate the purity of sorted cell populations, we analyzed *AIRE* gene expression (Figure 2G), typical of medullary TEC. As expected, the *AIRE* expression was higher in medullary TEC populations, with the subpopulation classified as cTEC exhibiting average expression close to zero. In fact, the data from three independent sorting pointed out that, on average, *AIRE* relative expression on mTEC is 29.45 times higher than in cTEC. There was no significant difference between samples from control and infected condition (*p* = 0.31), indicating that the infection by itself does not affect *AIRE* levels, although we have detected a significant interaction effect. This result suggests that the difference on *AIRE* expression due to TEC phenotype varies if there is infection (*p* = 0.04). Actually, the detected levels in TEC from infected animals where 13.45 times lower than samples from control mice.

### Changes in TEC-derived microRNA profiling in response to experimental chagas disease

**Figure 3:**
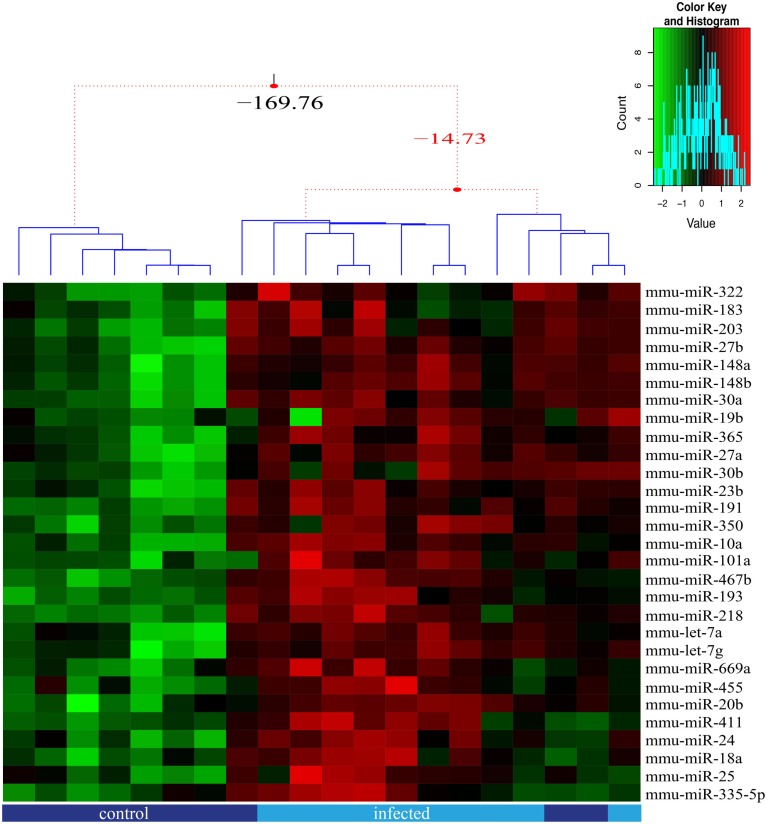
**miRNA relative expression in thymic epithelial cells from control and infected mice**. The miRNA expression of 85 miRNAs was analyzed for each sorted TEC population, in five replicates, with samples from control (dark blue) and infected (light blue) mice being paired. We present the 29 miRNAs that were significant (adjusted *p*-value ≤0.05) differentially expressed due to infection and the samples clustered according to expression profile similarity. Three groups of samples were identified based on their expression profile, as illustrated by the dendrogram.

We analyzed herein 85 miRNAs in order to approach putative molecular alterations in TEC following response to *T. cruzi* acute infection, and that might be related to the previously reported thymic atrophy and abnormal scape of immature thymocyte (20, 21, 32, 33). We found that 29 out of the 85 miRNAs were significantly differently expressed between TEC from infected and normal mice (adjusted *p* ≤ 0.05), all were up-regulated (Figure 3) whereas differences in further 13 miRNAs were suggestive (Figure S3 in Supplementary Material).

We also detected significant interaction effect for 9 miRNAs (adjusted *p*-value ≤0.05 FDR corrected), where the response to the infection differed according to the TEC phenotype (Figure 3; Figure S2 in Supplementary Material), indicating that the increase rate of miRNA expression is higher in cortical TEC.

Additionally, seven miRNAs exhibited a consistent pattern of no amplification in TEC from infected animals (miR-144, miR-208b, miR-291b-3p, miR-295, miR-302a, miR-488, and miR-654-3p, Figure S4 in Supplementary Material). These miRNA can target genes involved with TGF-β signaling pathway (Palu et al., unpublished data).

### *Trypanosoma cruzi* acute infection increases expression of miRNA known to modulate important biological processes

More than 60% of mammalian mRNAs are regulated by miRNA, whereas many can be targeted by more than one miRNA. Conversely, a single miRNA can have more than one target (34). Here, we identified miRNAs modulated in TEC due to *T. cruzi* infection, based on differential expression between infected and control mice.

**Figure 4:**
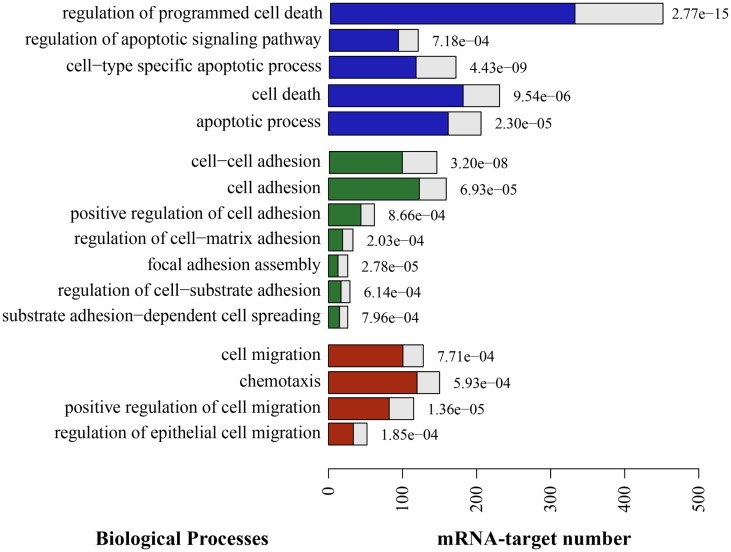
**Putative enriched biological processes modulated by TEC miRNA following *Trypanosoma cruzi* infection**. The putative mRNA targets for the 29 miRNAs that were differentially expressed due to infection are consistently involved in a long list of biological processes. Here, we show the most relevant processes in the context of cell death (blue), adhesion (green), and migration (red). The full length of the bars represent the total putative target mRNAs known to be involved in this process, in opposition to the colored bar inside, that represents the expected mRNA number. On the right side of each bar, there is the adjusted *p*-value indicating that the enrichment of those events is not by chance.

To approach the putative roles of these miRNAs, we identified potential targets using available algorithms. Yet, these algorithms usually predict hundreds of potential target genes for a single miRNA and often generate false-positive candidates. In order to reduce such a high number of theoretical targets, and to make a more reliable prediction, we applied five different algorithms, and considered as potential targets only those genes predicted by at least three of these algorithms. The results from miRNAs predicted targets analysis were then combined with GO-term enrichment analysis to identified biological processes over represented among the list of target genes, so that to identify miRNA associated biological functions. Significant enrichment of predicted targets revealed cell adhesion, cell migration, and cell death among others biological processes (Figure 4).

### Potential network among miRNAs and corresponding predicted targets

**Figure 5:**
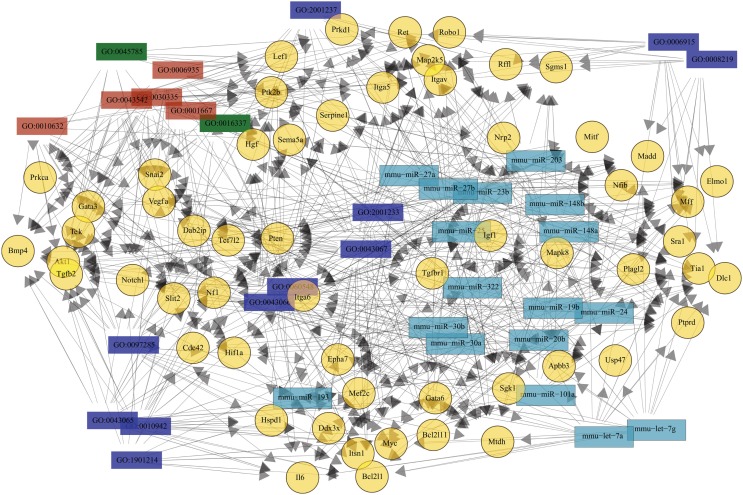
**Theoretical relationship among the most recurrent miRNA, mRNA, and biological processes**. The network of the 29 differentially expressed miRNAs and their targets is very complex. To illustrate this network, only molecules or processes related with at least 11 elements are shown. There are 17 miR differentially expressed due to infection (cyan) and their 58 putative targets (yellow), plus 18 biological processes related to it. Biological processes are identified by gene ontology (GO) tags, where blue represents cell death, red are cell adhesion, and green cell migration. The relationships are illustrated by the arrows.

Given the lack of data regarding TEC molecular pathways during infection, we evaluated *in silico* potential interaction network between 29 differentially expressed miRNAs and the predicted targets related to cell death, cell migration, and cell adhesion (Table S1 in Supplementary Material). The complexity of the relationships is shown in Figure 5, where the elements shown were selected based on having the minimum of 11 relations. All 17 miRNAs have at least one putative target related to the negative regulation of extrinsic apoptotic signaling (GO:2001237), a process that was also related to 12 out of the 58 illustrated genes. Nevertheless, among the genes involved in cell death, only *Bcl2l11* was exclusively related to positive regulation of cell death. These miRNAs could be targeting *Serpine1*, *Tgfbr1*, *Vegfa*, *Igf1*, *Hgf*, *Snai2*, *Rffl*, *Map2k5*, *Itgav*, and *Sgms1* mRNAs, which are related to inhibition of apoptotic externals signals.

Many putative targets (32 out of 56) were related to cell migration, whereas 18 were associated to chemotaxis. More interesting, the presence of 21 targets associated with positive regulation of cell migration suggests that miRNAs could be inhibiting molecules that promote migration. Among the 17 targets associated to adhesion, 8 are known to be involved in positive regulation (GO:0045785) and cell-cell adhesion (GO:0016337). The increase in miRNA targeting genes that favor adhesion may explain some of the described alterations in *T. cruzi* acutely infected mice.

## Discussion

The mechanisms by which TEC regulate thymic atrophy appear to be under the tight control of miRNAs (24). Here, we analyzed the miRNA expression in cortical and medullary TEC from *T. cruzi* acutely infected mice. Our results provide novel insights into the molecular regulation of TEC-associated thymic involution secondary to infection, using the experimental model of acute Chagas disease.

It has been previously shown that thymic involution, reduction on T cell output, increased susceptibility to autoimmune disease and loss of TEC numbers are associated with ablation of mature miRNAs (7). Although those are events similar to the ones observed in infected mice, it is noteworthy that the infection induced an upregulation of differentially expressed miRNAs in both cTEC and mTEC subsets, whereas in some cases the increase in expression was significantly higher only in cTEC, suggesting that the *T. cruzi* infection triggers different responses according to TEC phenotype.

Among miRNAs significantly modulated due infection, miR-27a and miR-27b, also exhibited dependence whether TEC sorted out from the thymus exhibited cortical or medullary phenotype. Mature miR-27a and miR-27b differ from each other by just one nucleotide and are transcribed from paralog clusters, the intergenic miR-23a~27a~24 cluster (localized in chromosome 9q22) and the intronic miR-23b~27b~24 cluster (localized in chromosome 19p13) (35, 36). Yet, there is limited information regarding the functions of this cluster in infectious diseases. Herein, we found an upregulation of the miR-23b~27b~24 cluster, thus at variance with the findings observed in primary macrophages, which exhibit rapid decrease miR-27a and miR-27b expression upon murine cytomegalovirus infection (37). Nevertheless, the *Cryptosporidium parvum* infection, a protozoan parasite that infects the gastrointestinal epithelium, causes miR-27b upregulation that suppresses KH-type splicing regulatory protein and contributed to epithelial production of NO, helping the epithelial antimicrobial defense (38). In *T. cruzi* infection, the serum levels of NO increase both in mice and humans (9, 39), and high intracardiac contents of NO synthase and NO metabolites have been detected (40). Interestingly, *T. cruzi* can infect TEC and, although only small fractions of TEC are invaded (13, 14), the presence of the parasite may trigger NO production.

Regarding the 23b~27b~24 cluster upregulation, miR-24 can be highlighted. It is known that mRNA-target for a particular miRNA depends of cell context and this is the case of miR-24, which has been described in apoptosis and cell survival (41–44). In cardiomyocytes, miR-24 directly targets the proapoptotic protein Bim and inhibits apoptosis. Moreover, *in vivo* delivery after myocardial infarction suppressed cardiac cell death and rescued cardiac dysfunction (42). Yet, miR-24 function is complex since it enhanced survival in myeloid and B cell lines, as well as primary hematopoietic cells (44). Importantly, it has been shown that miR-24 expression differed in age-related thymic involution. When comparing young *versus* aged TEC (a mix of cTEC and mTEC) a decrease in miR-148b, miR-19b, miR-24, and miR-322 expression was seen in aging (45). Herein, using *T. cruzi*-induced thymic atrophy, we showed an upregulation of these miRNAs in TEC. Both infection and aging-induced thymic involution are due to multifactorial events; in the aging case, we can highlight the sex hormone dependence and the increase in adipose tissue, whereas in infection, the immune inflammatory response and stress-related hormones are undoubtedly relevant (16, 46, 47). Nevertheless, it has yet to be established the tuned regulation of this miRNA in the various thymic atrophy induced situations.

It is important to take into account that changes in miRNA profile seen here are in consequence of the thymic stress brought out by the infection with activation of the immune system and the hypothalamus–pituitary–adrenal axis. This stress results in hormonal imbalance (high levels of GC) that affects intrathymic homeostasis promoting thymic atrophy by a massive depletion of immature CD4^+^CD8^+^ T cells and the export of immature thymocytes to periphery (9, 16, 17). Under stress conditions, miRNA can act as restorer to homeostasis or as an enforcer of new gene expression program so that to adapt to the new condition (34). An example is the regulatory action exerted by miR-10a and miR-182 upon Th1- or Th2-associated T regulatory cells, respectively, where CD4^+^Foxp3^+^ cells orchestrate distinct miRNA pathways in response to local environmental factors (48). Furthermore, miR-10a expression is stimulated by TGF-β, making it a good example of how environmental factors coordinate distinct miRNA pathways and regulates cell fate. In fact, TGF-β seems to be a molecular node of the infection since the gene encoding its receptor appears in the middle of our microRNA network (Figure 5), where the gene for TGF-β2 is also present. TGF-β is able to regulate CD4^−^CD8^−^ development through direct interaction with thymocytes but also by binding to TEC surface (49). Although, it is unclear if the increased miR-10a in TEC from infected mice is part of a host response due TGF-β enhancement or if miR-10a is a fine-tuning factor in TEC, our results suggest that TGF-β signaling is a key pathway in the thymic involution process. More studies will be necessary to define TGF-β role in TEC, but it has been already shown that the inhibition of this pathway decelerates the process of age-related thymic involution (50), therefore suggesting a common pathway between thymic involution due to senescence and infection.

Interestingly, GC also regulated miRNAs (51, 52). The systemic stress induced by dexamethasone intraperitoneal injection, a synthetic GC causes a significant loss of the CD4^+^CD8^+^ thymocytes within 24 h and a reduction in miR-17-92 cluster (miR-17, miR-20a, miR-20b, and miR-106a) in whole thymus samples (52). Although we were also studying miRNA expression in a condition where there is CD4^+^CD8^+^ cell loss, in TEC we observed an upregulation of miR-20b and a suggestive increase in miR-20a expression, suggesting that intrathymic regulation of miR-20b is cell type specific.

Glucocorticoids can also reduced the protein expression of Drosha’s co-factor DGCR8/Pasha and Dicer, two indispensable enzymes for miRNA bioprocessing pathway in thymocytes (51). Moreover, Dicer- and DGCR8-deficient mice are incapable to sustain proper thymic architecture and promote thymocyte development, with a severe loss of TEC, demonstrating the miRNA role in TEC maintenance and function (32, 33). In fact, in mice where TEC do not produce miRNA due conditionally inactivate *Dgcr8* gene, there is a specific loss of mature mTEC^hi^ and AIRE^+^ subsets that induce a breakdown in thymic central tolerance with the presence of autoantibodies or development of spontaneous autoimmunity (33). On the other hand, *AIRE* knockdown results in modulation of different miRNAs (53, 54), with upregulation of miR-20b, miR-191, and miR-411 (54), which is consistent with our observation. In our study, there was donwregulation of *AIRE* expression due infection, concomitant with upregulation of those miRNAs.

Abnormally release of potential autoreactive T cells from the thymus occurs in patients with severe clinical form of Chronic Chagas disease and also in mouse experimental model (19). Intriguingly, despite the thymic escape of T cells bearing “forbidden” T cell receptor that should be deleted by negative selection (21), some evidence points to normal promiscuous gene expression in infected thymuses suggesting that negative selection can induce tolerance. Indeed, the escape of CD4^+^CD8^+^ T cells to the periphery seems to be more related with a higher fibronectin-driven migration than defects in negative selection (18, 20, 21). Considering that TEC play a role in thymocyte migration and that we defined enhanced intrathymic fibronectin and laminin deposition in *T. cruzi* acutely infected mice (1), we performed biological processes GO-term enrichment analysis and evaluated *in silico* potential interaction network among the 29 differentially expressed miRNAs and their predicted targets. This network analysis predicted cell adhesion, regulation of cell–matrix adhesion, cell migration, chemotaxis, regulation of programed cell death, and apoptotic processes to be altered as a consequence of *T. cruzi* acute infection. Taking together, these data point out miRNA as candidates to orchestrate thymic atrophy from the TEC perspective, since the alteration herein studied precedes the involution.

The intrathymic T cell migration is a multivectorial process where each individual vector represents a given molecular interaction, as, for example, those interactions mediated by ECM. Accordingly, changes in the ECM contents should result in modulation of thymocyte migration (4, 20). Although there are studies in cancer (55), there is a lack of information concerning the role miRNAs in regulating ECM molecules in the thymus, and more particularly in TEC. We found correlations indicating putative intrathymic functions for some miRNAs, such as miR-183 that direct regulates integrin β1 expression (56), miR-143, suppressing fibronectin directly (57), miR-218 controlling focal adhesion kinase (58), and miR-203 increasing metalloproteinase-1 expression (59).

Overall, in this study, we show differentially expressed miRNAs in TEC from *T. cruzi* acutely infected mice, highlighting miRNAs as possible mediators of thymic atrophy. To our knowledge, this is the first study to show miRNA expression in TEC from infected mice. Further studies are needed to define the targets and dissect the role of TEC miRNAs in the context of infection.

## Author Contributions

LL-L and CP designed and performed the experiments, analyzed and interpreted the data, and wrote the manuscript; MR-A performed quantitative polymerase chain reaction analysis and the bioinformatics analyses; BP performed the cell sorting; AM contributed to cell sorting standardization and to writing the manuscript; MG-S and AC contributed to the microRNA assays; and WS conceived the project, provided the budget, and participated in writing the manuscript.

## Conflict of Interest Statement

The authors declare that the research was conducted in the absence of any commercial or financial relationships that could be construed as a potential conflict of interest.

## Supplementary Material

The Supplementary Material for this article can be found online at http://journal.frontiersin.org/article/10.3389/fimmu.2015.00428

Figure S1 **Selection of multiple internal control reference for normalization**. **(A)** Calculation of average expression stability values of five different internal control reference, including one spike-in (cel-miR-39), using geNorm (23). The geometric mean of five small RNAs (Rnu6, cel-miR-39, snoRNA251, snoRNA 142, and snoRNA 202) was used for normalization. Crossing point mean of the samples shows the level of expression for **(B)** cel-miR-39 **(C)**, snoRNA 142, and **(D)** snoRNA202.

Figure S2 **Significant differentially expressed miRNA in thymic epithelial cells from control and infected mice separated by TEC phenotype**. The miRNA expression of 85 miRNAs were analyzed for each sorted TEC subpopulation, in five replicates, where samples from control, cortical TEC (orange) and medullary TEC (dark blue) and infected, cortical infected TEC (yellow) and medullary infected TEC (light blue). We present the 29 miRNAs that were significantly differentially expressed (adjusted *p*-value ≤0.05) due to infection.

Figure S3 **Suggestive differentially expressed miRNA in thymic epithelial cells from control and infected mice separated by TEC phenotype**. The expression of 85 miRNAs was analyzed for each sorted TEC subpopulation, in five replicates, where samples from control, cortical TEC (orange) and medullary TEC (dark blue) and infected, cortical (yellow) and medullary TEC (light blue) are showed. We present the 13 miRNAs that were suggestive significance (adjusted *p*-value ≤0.1) differentially expressed due to infection.

Figure S4 **miRNAs with a consistent pattern of no amplification in TEC from infected animals**. The miRNA expression of 85 miRNAs was analyzed for each sorted TEC subpopulation, in five replicates, where samples from control, cortical TEC (orange) and medullary TEC (dark blue) and infected, cortical (yellow) and medullary TEC (light blue) are showed. We present the seven miRNAs that exhibited a consistent pattern of no amplification in TEC from infected animals, being clearly detected in control samples.

Table S1 **List of microRNAs and the respective putative mRNA targets**.

## Footnotes

^1^http://www.mirbase.org/ftp.shtml

^2^http://www.microrna.org/microrna/getDownloads.do

^3^ftp://mirnamap.mbc.nctu.edu.tw/miRNAMap2/miRNA_Targets/Mus_musculus/

^4^http://mirtarbase.mbc.nctu.edu.tw/php/download.php

^5^http://www.targetscan.org/cgi-bin/targetscan/data_download.cgi?db=mmu_61

^6^http://www.bioconductor.org/packages/release/bioc/html/Rgraphviz.html

## Abbreviations

AIRE, autoimmune regulator; ECM, extracellular matrix; pGE, promiscuous gene expression; TEC, thymic epithelial cells.
