# Supplementary figures and images for: Differential Expression of microRNAs in Thymic Epithelial Cells from Trypanosoma cruzi Acutely Infected Mice: Putative Role in Thymic Atrophy
Source: Front Immunol. 2015 Aug 21;6:428. doi: 10.3389/fimmu.2015.00428 (PMC4543887; doi:10.3389/fimmu.2015.00428)

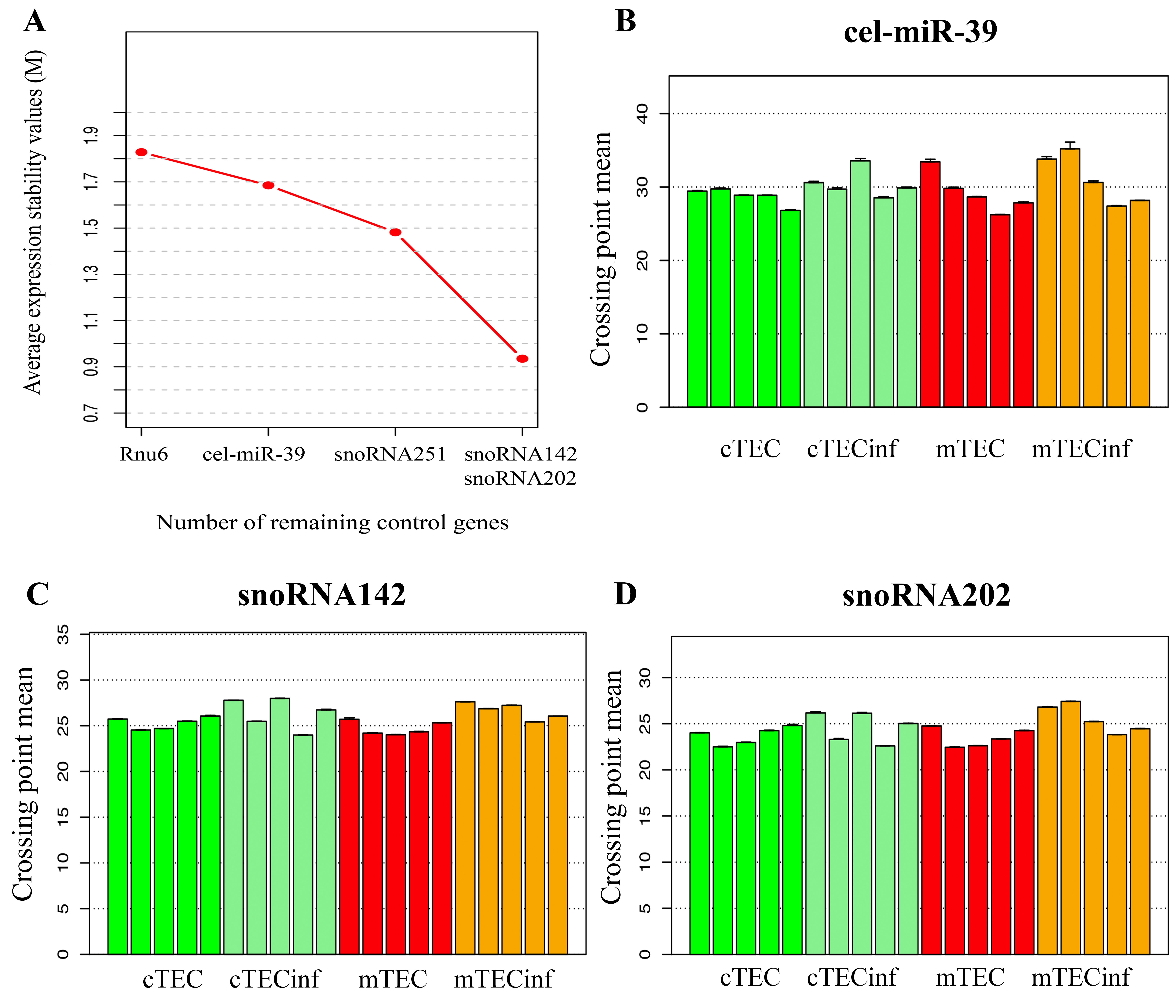

Supplement: Figure S1 — Selection of multiple internal control reference for normalization. (A) Calculation of average expression stability values of five different internal control reference, including one spike-in (cel-miR-39), using geNorm (23). The geometric mean of five small RNAs (Rnu6, cel-miR-39, snoRNA251, snoRNA 142, and snoRNA 202) was used for normalization. Crossing point mean of the samples shows the level of expression for (B) cel-miR-39 (C), snoRNA 142, and (D) snoRNA202. [file Image_1.TIF]

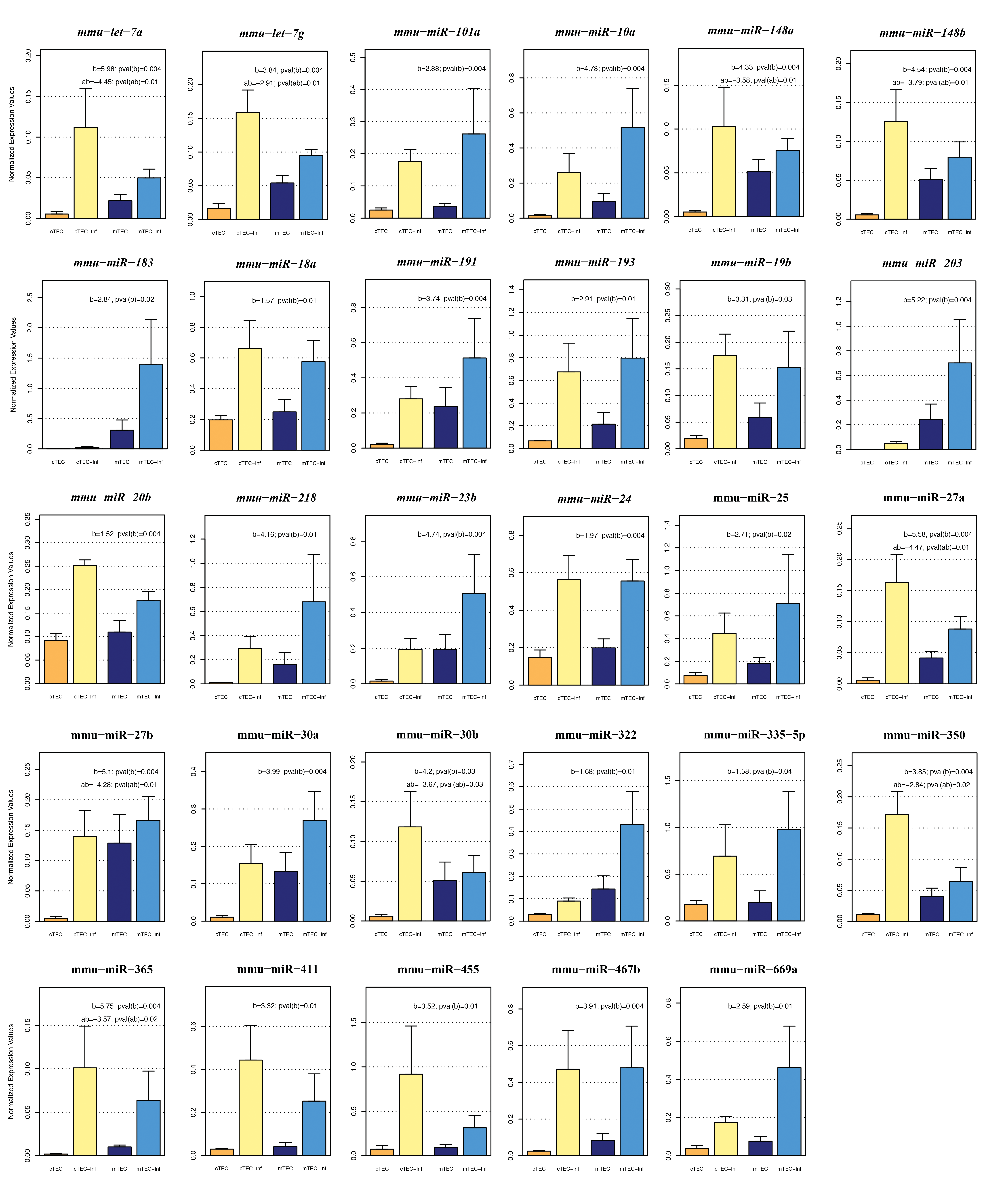

Supplement: Figure S2 — Significant differentially expressed miRNA in thymic epithelial cells from control and infected mice separated by TEC phenotype. The miRNA expression of 85 miRNAs were analyzed for each sorted TEC subpopulation, in five replicates, where samples from control, cortical TEC (orange) and medullary TEC (dark blue) and infected, cortical infected TEC (yellow) and medullary infected TEC (light blue). We present the 29 miRNAs that were significantly differentially expressed (adjusted p-value ≤0.05) due to infection. [file Image_2.TIF]

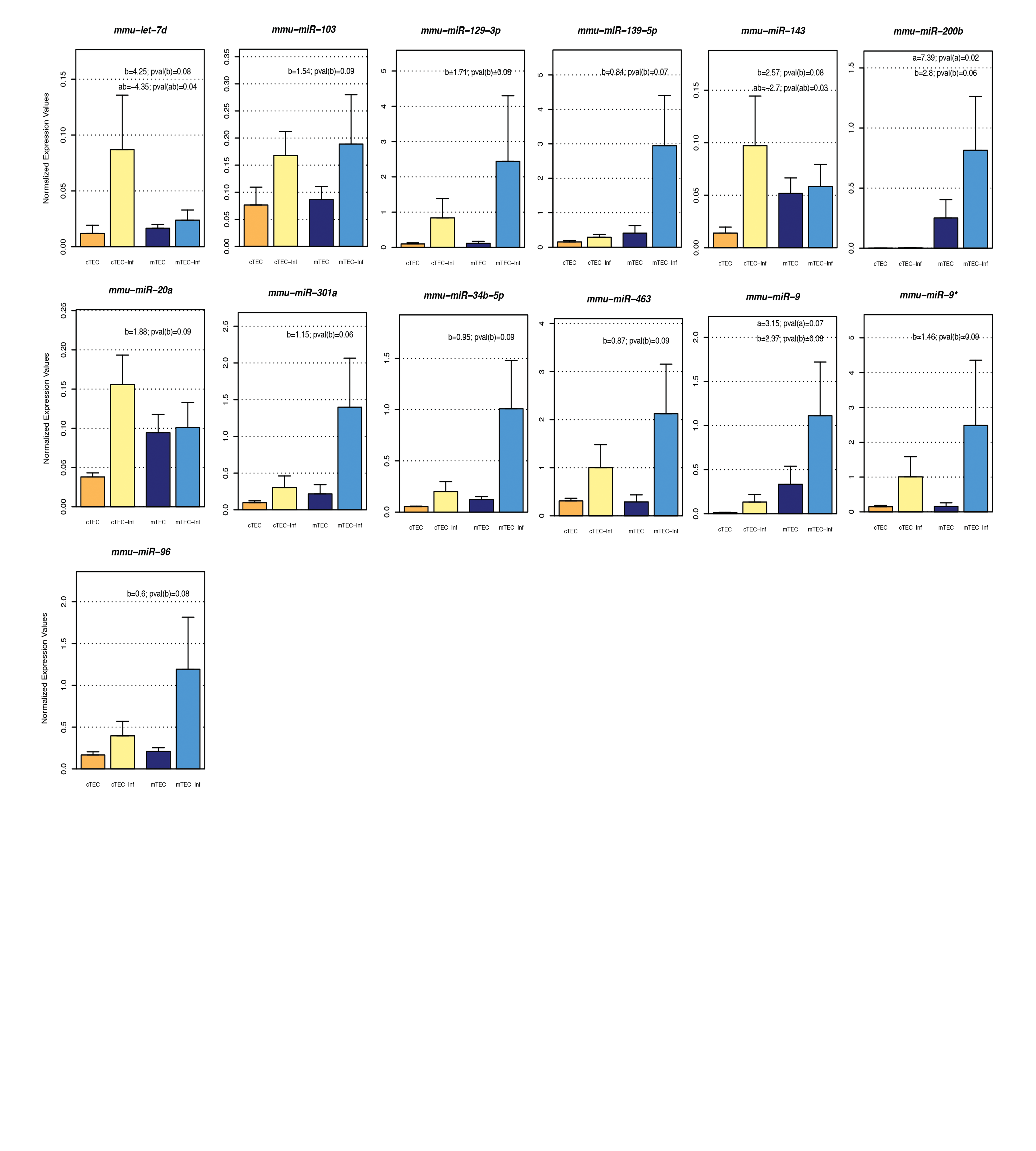

Supplement: Figure S3 — Suggestive differentially expressed miRNA in thymic epithelial cells from control and infected mice separated by TEC phenotype. The expression of 85 miRNAs was analyzed for each sorted TEC subpopulation, in five replicates, where samples from control, cortical TEC (orange) and medullary TEC (dark blue) and infected, cortical (yellow) and medullary TEC (light blue) are showed. We present the 13 miRNAs that were suggestive significance (adjusted p-value ≤0.1) differentially expressed due to infection. [file Image_3.TIF]

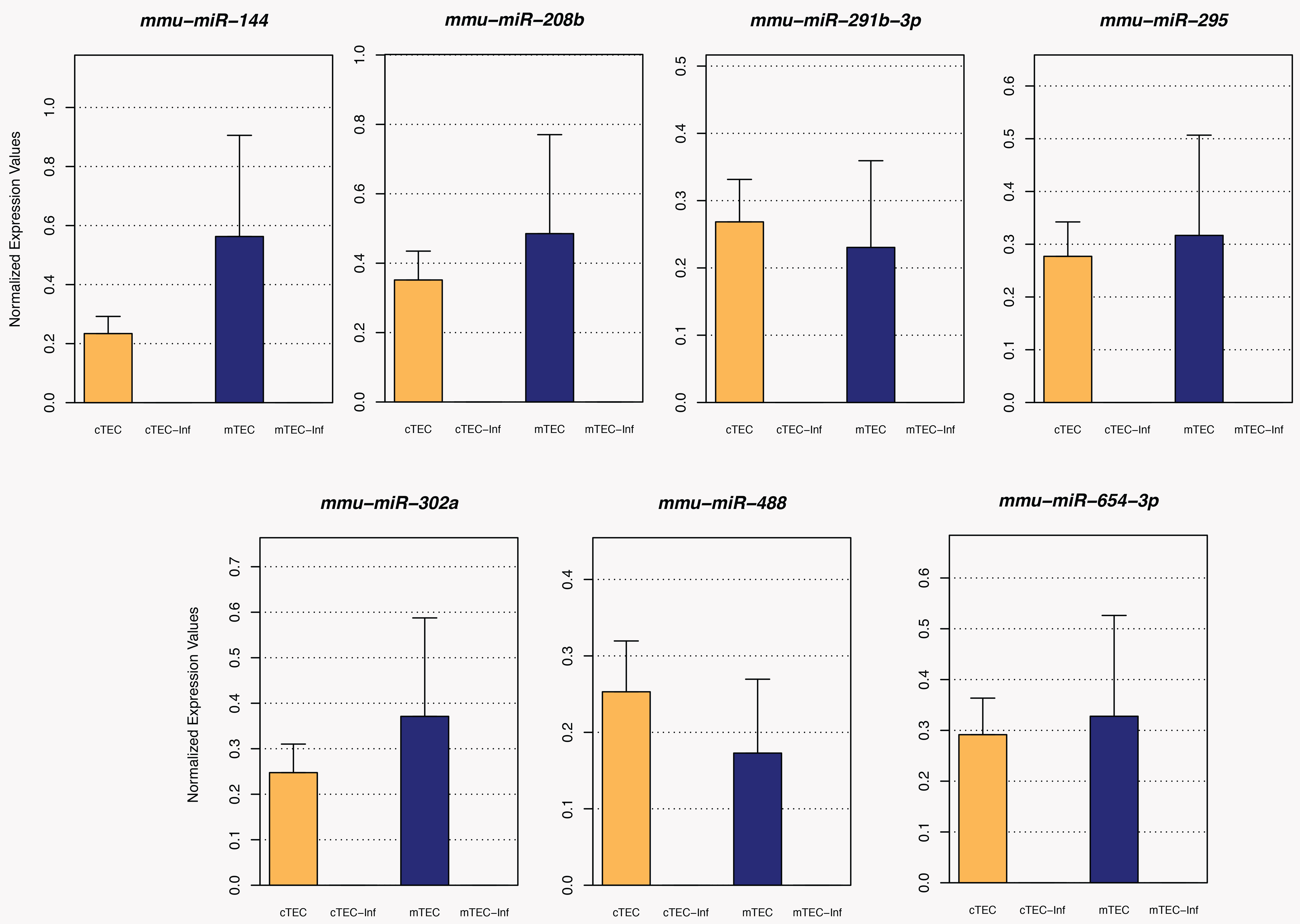

Supplement: Figure S4 — miRNAs with a consistent pattern of no amplification in TEC from infected animals. The miRNA expression of 85 miRNAs was analyzed for each sorted TEC subpopulation, in five replicates, where samples from control, cortical TEC (orange) and medullary TEC (dark blue) and infected, cortical (yellow) and medullary TEC (light blue) are showed. We present the seven miRNAs that exhibited a consistent pattern of no amplification in TEC from infected animals, being clearly detected in control samples. [file Image_4.TIF]
